# ^18^F-AlF-NOTA-octreotide PET/CT in the localization of tumor-induced osteomalacia: case series and literature review

**DOI:** 10.3389/fendo.2024.1400751

**Published:** 2024-06-03

**Authors:** Jing Li

**Affiliations:** Department of Nuclear Medicine, The Second Affiliated Hospital, Zhejiang University School of Medicine, Hangzhou, China

**Keywords:** 18F-AlF-NOTA-octreotide, PET/CT, hypophosphatemia, tumor-induced oseteomalacia, phosphaturic mesenchymal tumors

## Abstract

**Introduction:**

This study explores tumor-induced osteomalacia (TIO) through a case series and literature review, assessing the diagnostic potential of ^18^F-AlF-NOTA-octreotide (^18^F-OC) positron emission tomography/computed tomography (PET/CT).

**Methods:**

We analyzed TIO patients who underwent ^18^F-OC PET/CT. Parameters such as tumor dimension, the maximum standardized uptake value (SUVmax), the mean standardized uptake value (SUVmean) and metabolic tumor volume (MTV) were meticulously assessed. Clinical features and imaging characteristics pertinent to TIO were reviewed.

**Results:**

6 patients with clinical suspicion of TIO exhibited hypophosphatemia (0.25 to 0.64 mmol/L), elevated alkaline phosphatase (ALP) levels (142 to 506 U/L), and increased parathyroid hormone (PTH) levels (92.9 to 281.7 pg/mL). Of these patients, two underwent FGF-23 testing, with results of 3185.00 pg/ml and 17.56 pg/ml, respectively. Conventional imaging modalities depicted widespread osteoporosis, with several cases demonstrating fractures indicative of osteomalacic and associated pathological fractures. Subsequent ^18^F-OC PET/CT facilitated the accurate localization of causative tumors, with histopathological examination confirming the diagnosis of phosphaturic mesenchymal tumor (PMT). The interval from initial clinical presentation to definitive TIO diagnosis spanned approximately 2.5 years (range: 1 - 4 years), with tumors varying in size (maximum diameter: 7.8 to 40.0 mm), SUVmax (5.47 to 25.69), SUVmean (3.43 to 7.26), and MTV (1.27 to 18.59 cm^3^).

**Conclusion:**

The implementation of whole-body ^18^F-OC PET/CT imaging emerges as a critical tool in the identification of occult tumors causing TIO. Future investigations incorporating a broader cohort are imperative to further delineate the diagnostic and therapeutic implications of ^18^F-OC PET/CT in managing TIO.

## Introduction

Tumor-induced osteomalacia (TIO) is a rare paraneoplastic syndrome, primarily caused by the overproduction of phosphatonins, such as fibroblast growth factor 23 (FGF23), by mesenchymal tumors. The biochemical hallmark of TIO includes renal phosphate wasting-induced hypophosphatemia, elevated levels of parathyroid hormone (PTH) and alkaline phosphatase (ALP), inappropriately normal or frankly low 1, 25-dihydroxyvitamin D, and inappropriately normal or elevated FGF23 levels (1, 2). The surgical excision of the tumor represents the definitive therapeutic intervention, necessitating the precise detection and localization of the tumors as a prerequisite for the potential curative approach to TIO (3, 4). Nonetheless, these tumors frequently present asymptomatically, are diminutive in size, exhibit slow growth, and can be located anywhere within the body, from the skull to the feet, rendering the diagnostic process exceedingly challenging (5). This necessitates the employment of multimodal imaging techniques for accurate tumor localization. The general unfamiliarity with TIO among clinicians often leads to diagnostic inaccuracies and overlooked diagnoses. ^18^F-AlF-NOTA-octreotide (^18^F-OC) positron emission tomography/computed tomography (PET/CT) represents a novel molecular imaging modality, targeting somatostatin receptor-expressing tumors, and holds promise for application in evaluating neuroendocrine tumors. However, its efficacy in the assessment of TIO remains to be fully ascertained (6). This article delineates 6 cases of TIO accurately localized and pathologically diagnosed using ^18^F-OC PET/CT imaging, coupled with an analytical summary based on relevant literature to augment understanding of this condition.

## Methods

This study retrospectively collected and analyzed clinical and imaging data for 6 patients with suspected TIO, who underwent ^18^F-OC PET/CT imaging at the PET Center of the Second Affiliated Hospital of Zhejiang University School of Medicine between January 2022 and January 2024. All cases were pathologically confirmed as PMT. We gathered data on demographic details (gender and age), clinical manifestations, disease duration, tumor location, and serum levels of phosphate, ALP, PTH,1,25-dihydroxy vitamin D, and FGF23. Additionally, information on tumor biopsy and imaging findings from CT and MRI scans was compiled. The ^18^F-OC PET/CT examination data were processed using Siemens syngo software at the imaging station. PMTs typically occur in a wide range of locations, hence patients suspected of having PMTs usually require a PET/CT scan that covers the entire body from the skull to the feet. The tumor region of interest (ROI) was manually delineated, and with a threshold set at a standardized uptake value (SUV) of 2.5, the maximum standardized uptake value (SUVmax), the mean standardized uptake value (SUVmean), and metabolic tumor volume (MTV) were automatically calculated. Informed consent for the publication of case reports and associated images was obtained from all participating patients, who are currently under follow-up. The study received approval from the institutional ethics committee (approval number: 2023–1234).

## Results

### Case 1

**Figure 1:**
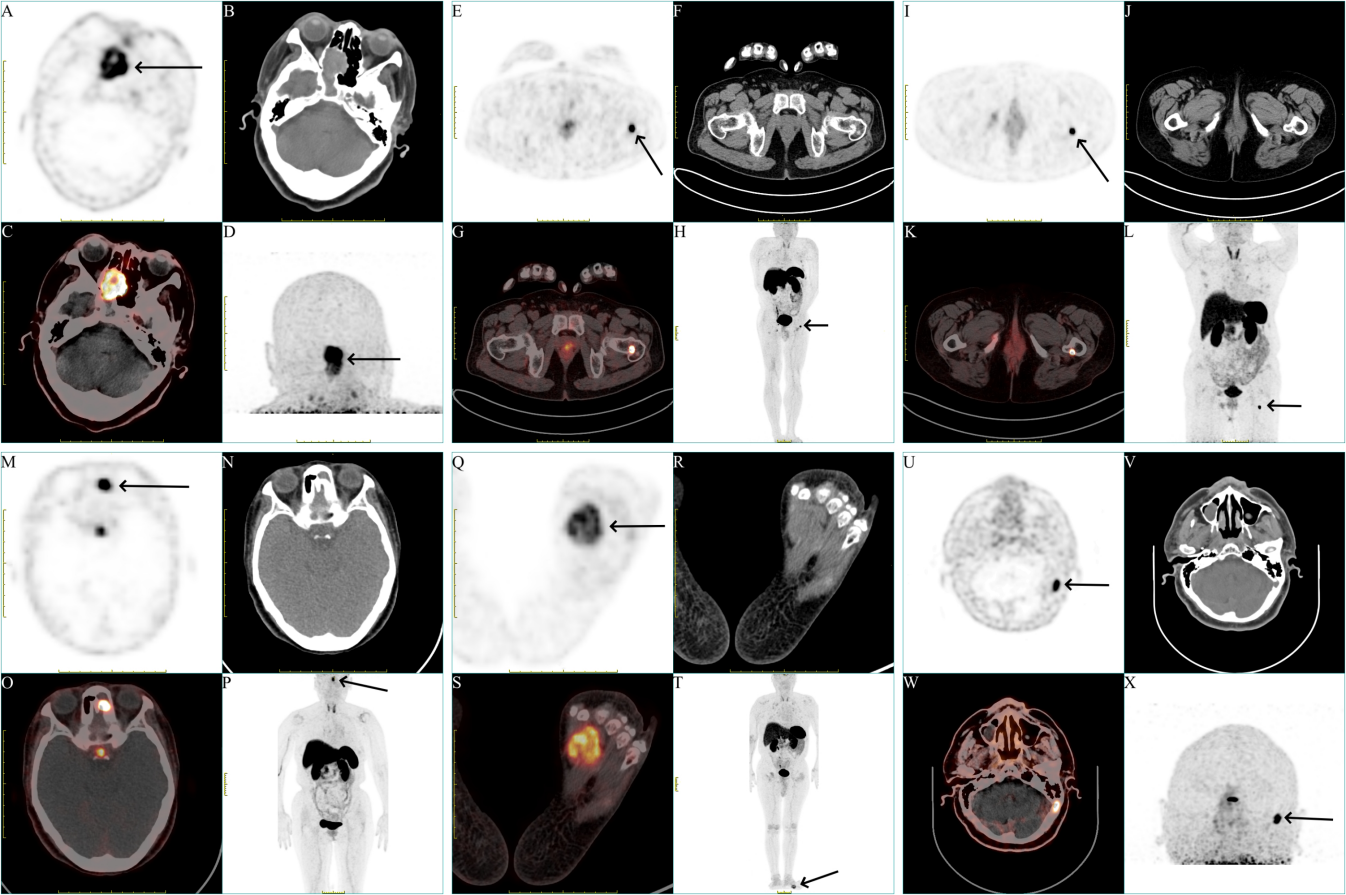
Representation of different locations of the tumors of all patients. **(A, E, I, M, Q, U)** Axial view of the tumors in ^18^F-OC PET (black arrow). **(B, F, J, N, R, V)** Axial view of the tumors in CT. **(C, G, K, O, S, W)** Fusion image. **(D, H, L, P, T, X)** Maximum-intensity projection (MIP) image of the patients (black arrow).

A 75-year-old woman presented with a two-year history of progressive polyarticular pain. An ^18^F-fluorodeoxyglucose (^18^F-FDG) PET/CT scan conducted one year prior identified multiple non-traumatic fractures across the right ilium, left femur, and bilateral pubic bones. Bone scintigraphy showed several areas of increased bone metabolism, initially suggestive of old fractures. Laboratory analyses revealed hypophosphatemia with serum phosphate levels ranging from 0.25 to 0.64 mmol/L (normal range, 0.85- 1.51 mmol/L), elevated ALP levels ranging from 173 to 257 U/L (normal range, 30- 120 U/L), and increased PTH at 104.8 pg/mL (normal range, 15- 65 pg/mL). Subsequent investigation with an ^18^F-OC PET/CT scan identified a soft tissue tumor (**Figures 1A–D**) measuring 27.0 × 22.0 mm in the right sphenoid sinus, characterized by a markedly high metabolic uptake (SUVmax = 14.24, SUVmean = 4.78, MTV = 18.59 cm^3^). Common metabolic parameters in PET imaging, such as SUVmax, SUVmean, and MTV, are crucial for assessing tumor diagnostics and therapy. A CT scan of the sphenoid sinus revealed a mass-like lesion with patchy high-density calcifications, well-defined margins, and partial osseous destruction of the ethmoid and sphenoid sinuses, extending to the medial wall of the right orbit. Contrast-enhanced MRI showed the lesion in the right nasal cavity-ethmoid sinus exhibiting heterogeneous T1 and T2 signals with uneven enhancement. Reviewing prior ^18^F-FDG PET/CT scans, the lesion was initially misinterpreted as inflammation due to its low metabolic uptake. An endoscopic biopsy of the sinonasal cavity was performed, with histopathology indicating a spindle cell tumor in the right nasal cavity. Immunohistochemical analysis revealed positive staining for Beta-catenin, BRG1, INI-1, CD34, H3K27me3, DOG-1, Cyclin D1, ERG, SSTR2, Vimentin, CD56, and SATB2, and negative for S-100, SOX10, STAT6, SMA, Desmin, CD99, CK(AE1/AE3), ER, PR, GFAP, EMA, P53, HMB45, and Melan-A. The proliferation index Ki-67 was noted at 15%.

### Case 2

A 44-year-old man presented with activity-related pain in his left hip joint, persisting for over three years. Initially diagnosed with rheumatoid arthritis, attributed to an elevated rheumatoid factor, the patient experienced symptomatic relief following treatment. Nonetheless, upon self-discontinuation of medication, the pain extended to the right hip joint, lumbar-sacral region, and bilateral intercosal areas. Bone density evaluation indicated osteoporosis. Laboratory investigation revealed a rheumatoid factor of 227.0 IU/ml (normal range, <19.5 IU/ml), hypophosphatemia with serum phosphate levels ranging from 0.46 to 0.52 mmol/L (normal range, 0.85- 1.51 mmol/L), elevated ALP levels ranging from 229 to 328 U/L (normal range, 30- 120 U/L), PTH at 92.88 pg/mL (normal range, 15- 65 pg/mL), and high-sensitivity C-reactive protein levels ranging from 12.7 to 17.6 mg/L (normal range, < 5 mg/L). An ^18^F-OC PET/CT scan revealed a quasi-circular, metabolically active bone tumor (**Figures 1E–H**) measuring 16.2 × 11.9 mm on the left femoral neck with significant metabolic uptake (SUVmax = 18.01, SUVmean = 6.16, MTV = 1.95 cm^3^). Hip joint CT scans displayed no discernible abnormalities. Contrast-enhanced MRI identified intertrochanteric sheet-like changes in the left femoral neck, exhibiting prolonged T1 and T2 signals and uneven enhancement, indicative of a potential left femoral neck base and intertrochanteric fracture, accompanied by surrounding bone marrow edema and localized granuloma proliferation. Subsequent left femoral tumor excision surgery was performed, with histopathological examination revealing spindle cell proliferation with mild atypia and a presence of basophilic material. Immunohistochemical analysis revealed positive staining for SATB2, and negative for H3.3G34W, CD68, SMA, Desmin, S-100, and CD34. The proliferation index Ki-67 was 1%. Remarkably, the patient’s serum phosphate levels normalized within one week post-operatively.

### Case 3

A 65-year-old woman presented with a two-year history of chronic hypophosphatemia and escalating bone pain. Bone densitometry revealed significant mass loss, documented as -1.4 at the right hip joint and -1.8 at the spine, unresponsive to calcium supplementation. Laboratory analyses revealed hypophosphatemia with serum phosphate levels ranging from 0.38 to 0.55 mmol/L (normal range, 0.85- 1.51 mmol/L), elevated ALP levels ranging from 166 to 206 U/L (normal range, 30- 120 U/L), elevated PTH levels ranging from 115.0 to 244.0 pg/mL (normal range, 15- 65 pg/mL), with a positive syphilis antibody. An ^18^F-FDG PET/CT scan conducted one month prior revealed multiple non-traumatic fractures in both ribs and the right pubic bone. Subsequent ^18^F-OC PET/CT scan identified a highly metabolic, quasi-circular bone tumor (**Figures 1I–L**) measuring 7.8 × 6.1 mm at the proximal left femur with significant uptake (SUVmax= 23.65, SUVmean = 7.13, MTV = 1.27 cm^3^). CT scans showed the tumor’s irregular shape and indistinct boundaries, involving the posterior cortex and subcortex with slight peripheral sclerosis, suggesting a potential tumor in the posterior segment of the left femoral proximal lesser trochanter. Enhanced MRI revealed abnormal signals beneath both the left femoral trochanter and the right pubic branch, characterized by long T1 and T2 signals, marked enhancement, and a pathological fracture beneath the right pubic branch. Reviewing the previous ^18^F-FDG PET/CT scans revealed a slight increase in metabolic activity at the proximal end of the left femoral (SUVmax = 4.27). The patient underwent surgical resection of the affected area at the left femoral proximal end, with pathology indicating clustered small round cell formation within the tumor. Immunohistochemical analysis revealed positive staining for Beta-catenin, SMA, CD34, CD56, ERG, SSTR2 and SATB2, and negative for Desmin, P53, S-100, SOX10, STAT6 and CK(AE1/AE3). The proliferation index Ki-67 was 5%.

### Case 4

A 64-year-old woman was admitted with a year-long history of general malaise and two months of untreated hypophosphatemia. Laboratory analyses revealed hypophosphatemia with serum phosphate levels ranging from 0.42 to 0.55 mmol/L (normal range, 0.85- 1.51 mmol/L), ALP at 142 U/L (normal range, 30- 120 U/L), and PTH at 281.7 pg/mL (normal range, 15- 65 pg/mL). An ^18^F-OC PET/CT scan detected a soft tissue tumor (**Figures 1M–P**) measuring 16.6 × 11.9 mm within the left nasal cavity and ethmoid sinus, showing abnormal metabolic uptake (SUVmax = 25.69, SUVmean = 7.26, MTV = 3.66 cm^3^). Enhanced CT imaging revealed the tumor’s presence across the left frontal sinus-anterior ethmoid sinus-maxillary sinus, characterized by uneven density and linear, punctate calcifications, with notable ring and strip enhancement post-contrast. MRI findings suggested inflammation in the left maxillary, frontal, and ethmoid sinuses, raising suspicions of a concurrent fungal infection. Focal resection of the left ethmoid sinus was performed, with pathology identifying a spindle cell lesion, chronic mucosal inflammation in the left ethmoid bulla, and a likely Aspergillus infection in the left maxillary sinus. Immunohistochemistry showed positivity for CD34, CK(AE1/AE3), CD56, SATB2, ERG, and SSTR2, and negative for S-100, SOX10, Beta-catenin, SMA, Desmin, Calponin, P63, STAT6, EMA, Syn, and PAX3(2q36). Postoperative management led to normalization of phosphate levels within two weeks, and PTH levels within 6 weeks.

### Case 5

A 53-year-old man developed left foot pain after a sprain, which was followed by widespread body pains, including the right foot, both hip joints, and the lumbar spine, over four years. Bilateral femoral neck fractures from an accidental fall five months prior showed no improvement after conservative treatment. Laboratory analyses revealed hypophosphatemia with serum phosphate levels ranging from 0.32 to 0.63 mmol/L (normal range, 0.85- 1.51 mmol/L), elevated ALP levels ranging from 442 to 506 U/L (normal range, 30- 120 U/L), elevated high-sensitivity C-reactive protein at 132.3 mg/L (normal range, < 5 mg/L), and FGF-23 at 3185.0 pg/ml (normal range, 23.3- 95.4 pg/ml). An ^18^F-OC PET/CT scan identified a significantly metabolically active soft tissue tumor (**Figures 1Q–T**) measuring 40.0 × 30.0 mm in the left foot with increased uptake (SUVmax = 5.47, SUVmean = 3.43, MTV = 11.82 cm^3^). CT imaging showed the mass with heterogeneous internal density. Surgical resection of the left foot tumor was performed, revealing proliferative ovoid cells with mild atypia, indistinct nucleoli, osteoclastic multinucleated giant cell reaction, hemorrhage, and hemosiderin deposition. Immunohistochemistry was positive for SSTR2, SATB2, CD56, CD163, CD68, and ERG, and negative for S-100, Syn, Desmin, P63, CD34, H3.3G34W, SMA, and CK(AE1/AE3). Despite two months of outpatient phosphorus supplementation, the patient remained hypophosphatemic, with persistent elevated ALP, PTH, and reduced FGF-23 levels. Nodular enhancement in the surgical area, observed via CT and MRI, suggested tumor recurrence, prompting recommendations for a second surgery intervention.

### Case 6

A 60-year-old man presented with a three-year history of unexplained weakness in both lower limbs, progressively exacerbated by pain in the knees and feet during ambulation. Laboratory investigations revealed hypophosphatemia with serum phosphate levels ranging from 0.30 to 0.46 mmol/L (normal range, 0.85- 1.51 mmol/L), elevated ALP levels ranging from 366 to 463 U/L (normal range, 30- 120 U/L), FGF-23 at 17.56 pg/ml (normal range, 23.3- 95.4 pg/ml) and total procollagen type 1 amino-terminal propeptide (tP1NP) at 92.37 ng/ml (normal range, 15.1- 58.6 ng/ml). An ^18^F-OC PET/CT scan disclosed osseous destruction (**Figures 1U–X**) measuring 17.0 × 10.0 mm on the left side of the occipital bone, displaying significantly increased metabolic activity (SUVmax = 13.15, SUVmean = 5.69, MTV = 1.31 cm^3^). Corroborated by MRI, the lesion suggested a benign or low-grade malignant tumor base on T1 isotensity, and T2 hyperintensity, and significant enhancement. Histopathological examination post-resection of the left occipital skull lesion revealed a benign vascular-rich spindle cell neoplasm with mild atypia. Immunohistochemical staining was positive for SATB2, FLI1, and CD34, and negative for STAT6, H3.3G34W, CD99, S-100, Desmin, and SMA. The proliferation index Ki-67 was 5%. Postoperatively, the patient’s serum phosphate levels normalized by the fifth day.

## Discussion

Hypophosphatemic osteomalacia (HO) consists of hypophosphatemia, elevated ALP, muscular weakness, and bone pain. Misdiagnosis of HO is common, with an average delay of about 5 years before diagnosis (7). It is often caused by severe vitamin D deficiency, hereditary hypophosphatemic rickets syndromes, renal tubular acidosis, primary hyperparathyroidism, medications, and TIO (8). TIO-related tumors primarily originate from mesenchymal tissues, mostly located in bone and soft tissue (5, 9). Zhang et al. (10) reported that among 135 cases, 54.5% of the causative tumors originated from bone, predominantly in the femur and jawbone, while 45.5% originated from soft tissues, located in areas such as the nasal cavity, sinuses, and periosteum. The most common pathological type is PMT, among others like giant cell tumor, prostate cancer, B-cell non-Hodgkin lymphoma, vasopericytoma, neurofibroma, hemangiopericytomas and osteofibrous dysplasia, etc (5, 7, 11–15). In 1947, McCance (16). reported the first case of osteomalacia caused by a femoral tumor, but the relationship between the tumor and osteomalacia was not clear. In 1959, Prader et al. (17) first elucidated the relationship between mesenchymal tumors and osteomalacia. In 1987, Weidner and Santa (18). coined the term ‘phosphaturic mesenchymal tumor-mixed connective tissue variant (PMTMCT)’ for osteomalacia-associated mesenchymal tumors. In 2004, Folpe et al. (19) first proposed classifying the mesenchymal tumor that causes TIO as an independent pathological hisological type, and it was included in the WHO (2013) Classification of Soft Tissue Tumors in 2013 (20).

Despite pathologic confirmation, locating TIO tumors is challenging due to their small size and indolence, requiring advanced imaging such as CT, whole-body MRI, bone scintigraphy, and ^18^F-FDG PET/CT (21–23). Conventional anatomical imaging modalities exhibit limited diagnostic accuracy for TIO and typically rely on the guidance provided by functional imaging techniques (24). In 1996, Reubi et al. (25) reported that a variety of mesenchymal-derived tumors express somatostatin receptor (SSTR), especially SSTR2 receptors, establishing SSTR imaging as a pivotal method for identifying causative tumors (26). Agents for single-photon imaging include ^111^In and ^99m^Tc-labeled SSTR (27–29). Positron imaging agents primarily consist of ^68^Ga-labeled SSTR octreotide variants (27, 30, 31). Particularly, ^68^Ga-DOTA-TATE, with stronger affinity to SSTR2 receptors, has become the preferred imaging choice for detecting causing tumors (32, 33). Zhang et al. (34) reported that in a study of 56 patients suspected of having TIO, the ^68^Ga-DOTA-TATE PET/CT scans demonstrated a sensitivity of 95.13%, and a specificity of 60.00% for detecting TIO. In another study by Zhang et al. (10) involving 159 patients suspected of TIO, the ^68^Ga-DOTA-TATE PET/CT scans achieved a sensitivity of 99.3% (134/135), a specificity of 79.2% (19/24), and an accuracy of 96.2% (153/159) in identifying TIO. These findings highlight the high efficacy and potential of ^68^Ga-DOTA-TATE PET/CT in accurately diagnosing TIO in suspected cases. However, the limited production capacity of ^68^Ga through germanium-gallium generators and the challenges associated with post-elution labeling, with a half-life of 68 minutes, has hindered the widespread use of ^68^Ga-labeled PET tracers, prompting the exploration of other radiotracer for SSTR imaging (35). ^18^F, a radionuclide commonly employed in clinical PET imaging, can be produced in bulk by cyclotrons, featuring a relatively longer half-life, thereby offering superior inherent resolution (36). In 2010, Laverman et al. (37, 38) presented a labeling method for ^18^F-OC and demonstrated its high *in vitro* binding affinity to SSTR in a preclinical model. In 2019, Long et al. (6) reported their initial clinical experience with ^18^F-OC in three healthy volunteers and twenty-two patients with neuroendocrine neoplasms (NENs). The tracer was proven safe with favorable pharmacokinetics and biodistribution characteristics, offering good detection of tumors with high tumor-to-background ratios. The application of ^18^F-OC PET/CT has significantly impacted the clinical diagnosis of neuroendocrine tumors (39–41), marking it as a valuable molecular imaging tool for identifying tumors expressing SSTR. Nonetheless, the use of ^18^F-OC PET/CT for the diagnosis and treatment of TIO remains limited by the small number of cases studied (42).

This study details the imaging characteristics of identifying TIO using ^18^F-OC PET/CT, underscoring the technique’s proficiency in locating elusive, solitary tumors predominantly found in the lower extremities. These slow-growing tumors, often small in size, can nonetheless provoke systemic symptoms. Our study revealed that among the 6 causative tumors identified, three originated from soft tissue and remaining three from bones, with soft tissue tumors presenting larger volumes than their bony counterparts. Notably, the smallest detected TIO tumor, overlooked by both conventional imaging modalities and ^18^F-FDG PET/CT, was successfully identified by ^18^F-OC PET/CT. This tumor, nestled in the femoral neck, measured just 1.27 cm^3^ in volume and 7.8 mm in maximum diameter. This finding supports the notion that the majority of TIO tumors are benign, non-invasive growths confined within bone structures. The study emphasizes that ^18^F-OC PET/CT, offering whole-body tomographic imaging from the skull to the feet, is crucial for patients suspected of having TIO, as it can detect tumors that may be missed by conventional imaging techniques focused on specific locations. Moreover, ^18^F-OC, serving as a targeted imaging agent, demonstrated higher metabolic uptake than nonspecific agents like ^18^F-FDG in TIO tumors, highlighting its utility in detecting tumors with increased SSTR expression. SUVmax and SUVmean are commonly employed to discriminate between malignant tumors and benign lesions, offering insights into the malignancy gradient of the tumor. Meanwhile, MTV is frequently employed to ascertain the metabolic volume of diseases exhibiting high uptake. These three metabolic parameters, ubiquitous in PET imaging, play a critical role in tumor diagnosis and treatment (43). Within this study, ^18^F-OC PET/CT identified tumors with SSTR overexpression, with SUVmax ranging from 5.47 to 25.69, SUVmean ranging from 3.43 to 7.26, and MTV ranging from 1.27 to 18.59 cm^3^. Additionally, the CT component aided in the localization of these tumors, particularly those that might be overlooked due to low metabolic uptake. Despite the benign nature of TIO tumors, there have been instances of malignancy, underscoring the necessity of complete surgical removal for a curative outcome, albeit with a risk of recurrence (44, 45). The study concludes with a follow-up on a patient who experienced tumor recurrence post-surgery, highlighting the importance of ongoing monitoring after treatment.

## Conclusion

Whole-body ^18^F-OC PET/CT imaging emerges as a groundbreaking SSTR-specific technique pivotal in pinpointing TIO. However, the study acknowledges the limitation posed by its small sample size, advocating for future research encompassing a larger cohort to refine the diagnostic accuracy of ^18^F-OC PET/CT for TIO. Such research would furnish clinicians with a more comprehensive diagnostic tool, potentially enhancing patient care, treatment strategies, and prognostic assessments.

## Ethics statement

Written informed consent was obtained from a legally authorized representative(s) for anonymized patient information to be published in this article.

## Author contributions

JL: Writing – original draft, Writing – review & editing.
